# Three-dimensional alignment of density maps in cryo-electron microscopy

**DOI:** 10.1017/S2633903X23000089

**Published:** 2023-03-10

**Authors:** Yael Harpaz, Yoel Shkolnisky

**Affiliations:** Department of Applied Mathematics, School of Mathematical Sciences, Tel-Aviv University, Tel-Aviv, Israel

**Keywords:** Cryo-electron microscopy, global map alignment, synchronization, three-dimensional alignment

## Abstract

A common task in cryo-electron microscopy data processing is to compare three-dimensional density maps of macromolecules. In this paper, we propose an algorithm for aligning three-dimensional density maps, which exploits common lines between projection images of the maps. The algorithm is fully automatic and handles rotations, reflections (handedness), and translations between the maps. In addition, the algorithm is applicable to any type of molecular symmetry without requiring any information regarding the symmetry of the maps. We evaluate our alignment algorithm on publicly available density maps, demonstrating its accuracy and efficiency. The algorithm is available at https://github.com/ShkolniskyLab/emalign.

## Impact Statement

This paper describes a fast algorithm for the rotational and translational alignment of three-dimensional density maps. Such alignment is an essential step in cryo-electron microscopy data processing. The algorithm is available at https://github.com/ShkolniskyLab/emalign.

## 1. Introduction

Single particle cryo-electron microscopy (cryo-EM) is a method to determine the three-dimensional structure of biological macromolecules from their two-dimensional projection images acquired by an electron microscope^(^^1^ ^)^. In this method, a sample of identical copies of the investigated molecule is quickly frozen in a thin layer of ice, where each copy is frozen at an unknown random orientation. The frozen sample is imaged by an electron microscope, resulting in two-dimensional images, where each image is a tomographic projection of one of the randomly oriented copies in the ice layer. The goal of single particle cryo-EM is to determine the three-dimensional structure of the molecule from the acquired two-dimensional images. A common task in cryo-EM data processing is to compare two density maps of the same molecule. This is required, for example, to estimate the resolution of the maps, evaluating their Fourier shell correlation curve^(^^2^ ^)^, or to analyze their different conformations. All these tasks require to first align two density maps, that is, to orient them in the same way in a common coordinate system. Due to the nature of the cryo-EM imaging process, the two density maps may differ not only in their three-dimensional orientation (i.e., their “rotation”) but may also have different handedness (namely, reflected relative to each other) and may be centered differently with respect to a common coordinate system.

In this paper, we propose an algorithm for aligning two density maps, which is fully automatic and can handle rotations, translations, and reflections between the maps. The algorithm requires as an input only the two density maps. In particular, it does not assume knowledge of any other information such as the symmetry of the maps.

Formally, let 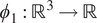 and 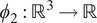 be two volumes such that
(1)

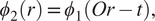

where 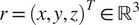 , 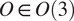 and 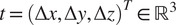 ( 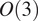 is the group of all orthogonal transformations of the three-dimensional space, namely rotations and reflections). The alignment problem is to estimate 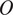 and 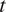 given 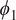 and 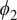 . The matrix 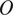 is known as the orientation parameter, and the vector 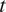 as the translation parameter. In practice, we only get samples of 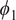 and 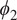 , arranged as three-dimensional arrays of size 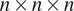 , where 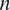 is the resolution of sampling. In cryo-EM, 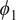 and 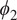 represent two reconstructions of the same underlying molecule that we would like to compare (such as two half maps from a refinement process). In principle, it is possible to approximate the solution to the alignment problem using exhaustive search, by generating a set of candidate pairs 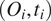 , where 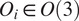 and 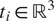 , and finding the pair which “best aligns” 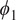 to 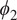 in some chosen metric. The purpose of the alignment algorithm presented in this paper is to estimate the optimal alignment parameters in a fast and accurate way.

The paper is organized as follows. In Section 2, we review existing alignment algorithms. In Section 3, we give a high level simplified description of our algorithm. A detailed description is then given in Section 4. This description relies on a method for aligning a single projection image against a volume, a procedure which is described in Section 5. In Section 6, we discuss implementation considerations of the algorithm and analyze its complexity. An optional procedure for refining the estimated alignment parameters is described in Section 7. In Section 8, we demonstrate numerically the properties and performance of our algorithm. Finally, in Section 9, we discuss the properties and advantages of our algorithm.

## 2. Existing Methods

There exist several methods for three-dimensional alignment of molecular volumes. The Chimera software^(^^3^ ^)^ offers a semi-automatic alignment method which requires the user to approximately align the volumes manually and then refines this alignment using an optimization procedure. This means that a sufficiently accurate initial approximation for the alignment is required. Achieving this initial approximate alignment manually naturally takes time and effort, yet it is crucial for the success of Chimera’s alignment algorithm. The alignment procedure implemented by Chimera maximizes the correlation or overlap function between the two volumes by using a steepest descent optimization. The iterations of this optimization stop after reaching convergence or after 2,000 steps.

Another alignment method is the projection-based volume alignment (PBVA) algorithm^(^^4^ ^)^. This method aligns a target volume to a reference volume by aligning multiple projections of the reference volume to the target volume whose orientation is unknown. The PBVA algorithm is based on finding two identical projections, a projection 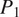 from the reference volume and a projection 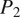 from the target volume as follows. The reference volume is projected at some known Euler angles, resulting in a projection 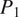 , and the matching projection 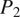 is found by maximizing the cross-correlation function between 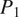 and a set of projections representing the possible projections of the target volume. The cross-correlation function is of five parameters—three Euler angles and two translation parameters in the plane of the projection 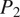 ^(^^5^ ^)^. Finally, the rotation between the volumes is estimated from the relation between the Euler angles corresponding to the projections 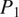 and 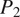 . After estimating the rotation between the volumes, the translation between them is found using projection images from the target volume. The translation between the volumes is estimated by least-squares regression using the two translation parameters of each projection from the target volume, where a minimum of two projections is required for calculating the three-dimensional translation vector. Using multiple projection images to estimate the translation between the volumes makes the alignment more robust.

The Xmipp software package^(^^6^ ^)^ also offers a three-dimensional alignment algorithm. It is based on expanding the two volumes using spherical harmonics followed by computing the cross-correlation function between the two spherical harmonics expansions representing the volumes^(^^7^ ^)^. The process of expanding a volume into spherical harmonics is called the spherical Fourier transform (SFT) of the volume, where like the fast Fourier transform (FFT) algorithm, there exists an efficient algorithm for calculating the SFT^(^^7^ ^)^. The process of calculating the cross-correlation function between the two spherical harmonics expansions of the volumes and estimating the rotation between the two volumes is implemented by a fast rotational matching (FRM) algorithm^(^^8^ ^)^. After estimating the rotation between the two volumes, the translation between them is found by using the phase correlation algorithm^(^^9^ ^)^.

Finally, the EMAN2 software package^(^^10^ ^)^ offers 2 three-dimensional alignment algorithms. In the first algorithm (implemented by the program e2align3d, now mostly obsolete), the rotation between the volumes is estimated using an exhaustive search for the three Euler angles of the rotation. First, the algorithm generates a set of candidate Euler angles with large angular increments. Then, the algorithm iteratively decreases the angular increments in the set of candidates in order to refine the resolution of the angular search^(^^11^ ^)^. A much faster tree-based algorithm is implemented in the program e2proc3d. This method performs three-dimensional rotational and translational alignment using a hierarchical method with gradually decreasing downsampling in Fourier space. In Section 8, we compare our algorithm to this latter algorithm, as well as to the FRM algorithm implemented by Xmipp.

## 3. Outline of the Approach

We are given two volumes 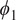 and 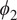 satisfying (1). For simplicity, we assume for now that the volumes have no symmetry and are related by rotation only (no translation nor reflection). We generate a projection image from 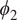 , denoted 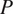 , corresponding to an orientation given by a rotation matrix 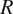 . Since 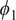 and 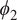 are the same volume up to rotation, we can orient 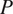 relative to 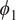 , that is, we can find the rotation 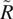 such that projecting 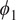 in the orientation determined by 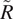 results in the image 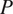 . As we show below, it holds that 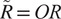 , where 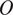 is the transformation from (1). Since 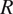 and 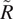 are known, we can estimate 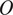 as 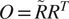 .

In practice, it may be that 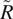 is not determined uniquely by 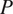 , as for example, a volume may have two very similar views even if it is not symmetric. Moreover, the volumes to align are discretized and sometimes noisy, which introduces inaccuracies into the estimation of 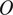 . Thus, to estimate 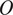 more robustly, instead of using a single image 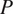 , we generate from 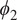 multiple images 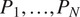 with orientations 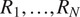 , align each 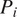 to 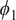 as above, resulting in estimates for 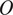 given by 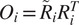 , and then estimate 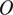 from all 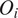 simultaneously by solving.

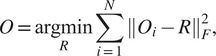

where 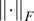 is the Frobenius matrix norm. In Section 4, we give an explicit solution for the latter optimization problem.

**Figure 1.**
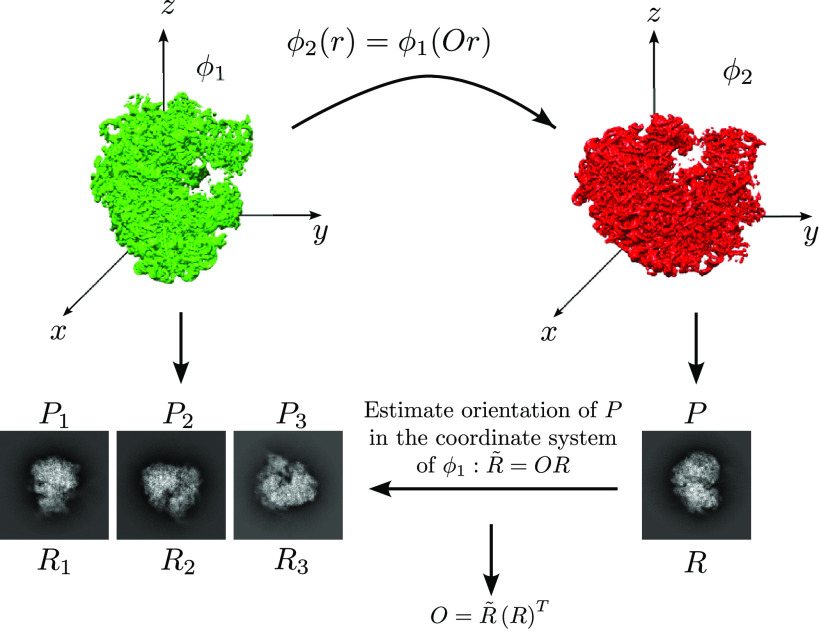
Outline of the algorithm.

The key of the above procedure is estimating the orientation of a projection image 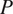 of 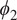 in the coordinate system of 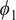 . This is done by inspecting a large enough set of candidate rotations and finding the rotation 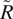 for which the induced common lines between 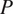 (when assuming its orientation is 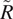 ) and a set of projections generated from 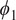 best agree. As inspecting each candidate rotation involves only one-dimensional operations (even if the input volumes are centered differently), it is very fast and highly parallelizable. Thus, this somewhat brute force approach is applicable to very large sets of candidate rotations (several thousands, for accurate alignment) and still results in a fast algorithm. We discuss below the complexity and advantages of this approach. We summarize the outline of our approach in Figure 1 and describe it in detail in Sections 4 and 5.

In the above approach, we assume that 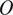 is a rotation. However, 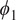 and 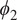 may have a different handedness, and so 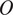 may include a reflection. The above approach can obviously be used to resolve the handedness by aligning 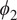 to 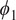 and to a reflected copy of 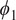 , and determining whether a reflection is needed using some quality score of the alignment parameters (e.g., the correlation between the aligned volumes). However, as we show below, in our method, there is no need to actually align 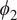 to a reflected copy of 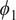 , saving roughly half of the computations (those required to actually align 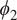 to a reflected copy of 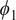 ), as explained in Section 4.

We next explain in detail the various steps of our algorithm, including handling translations, reflections, and symmetry in the volumes.

## 4. Estimating the Alignment Parameters

Consider two volumes 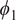 and 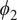 , where one volume is a rotated copy of the other (assuming for now no reflection nor translation between the volumes), namely (see (1))
(2)

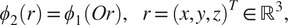

where 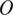 is an unknown rotation matrix. Our goal is to find an estimate for 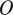 .

In case where 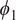 and 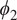 exhibit symmetry, the solution for 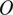 is not unique. To be concrete, we denote by 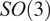 the group of all 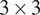 rotation matrices. A group 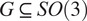 is a symmetry group of a volume 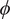 , if for all 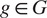 it holds that
(3)

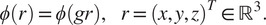

In other words, a symmetry group of a volume is a group of rotations under which the volume is invariant (see^(^^12^ ^)^ for more details). If we denote the symmetry group of 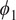 by 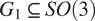 and define 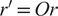 , then, from (2) and (3), we get for any symmetry element 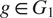



(4)

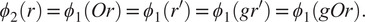

Comparing the latter with (2), we conclude that the solution for 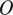 is not unique, and we thus replace the goal of finding 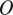 by finding any 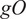 for some arbitrary element 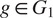 of the symmetry group.

Note that we assume that 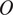 is a rotation, namely that 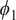 and 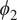 are related by rotation without reflection. The case where 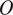 is a reflection will be considered below. Let 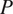 be a projection image generated from 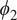 using a rotation 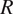 , that is,
(5)

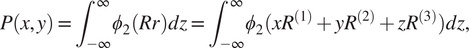

where 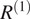 , 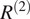 , 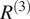 are the columns of the matrix 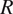 and 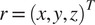 . From (2), we have that
(6)

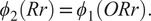

Thus, using (5), we have
(7)

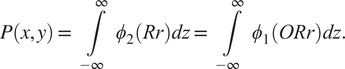

Equation (7) implies that if 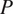 has orientation 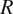 with respect to 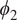 , then it has orientation 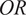 with respect to 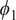 . In Section 5, we describe how to estimate 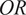 given 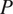 and 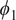 , namely how to estimate a rotation 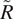 that satisfies 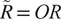 . If the volume 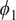 is symmetric with symmetry group 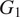 , then (as shown above) the rotation 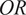 is equivalent to the rotation 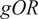 for any 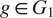 , and moreover, the two rotations cannot be distinguished. Thus, we conclude that

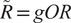

for some unknown 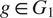 . Using the latter equation, we can estimate 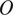 as
(8)

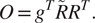

Note that in the latter equation 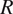 is known, 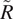 can be estimated using the algorithm in Section 5 below, and 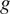 can be arbitrary. Thus, (8) provides a means for estimating 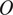 .

However, to estimate 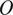 more robustly, we use multiple projections generated from 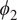 . Let 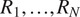 be random rotations, and let 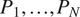 be the corresponding projections generated from 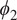 according to (5). Using the procedure described above, we estimate for each 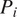 a rotation 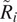 that satisfies 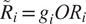 for some unknown 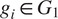 . Thus, as in (8), we can estimate 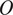 using any 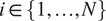 by
(9)

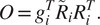

Contrary to (8), if we want the right-hand side of (9) to result in the same 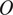 for all 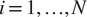 , then 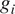 cannot be arbitrary. In order to estimate 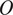 , we therefore need to find 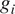 , 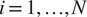 and combine all estimates for 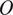 given in (9) into a single estimate.

To that end, define
(10)

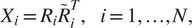

and look at the matrix 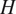 of size 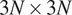 whose 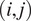 block of size 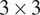 is given by (see (9) and (10))
(11)

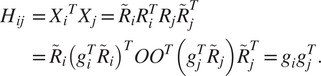

By a direct calculation, we get that the matrix of size 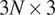



(12)

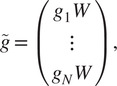

where 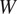 is an arbitrary 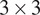 orthogonal matrix (i.e., 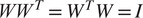 ), satisfies
(13)

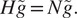

 Equation (11) also shows that the matrix 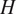 is of rank 3, which together with (13) implies that 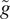 can be calculated by arranging the three leading eigenvectors 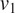 , 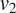 , 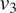 of 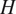 in a matrix
(14)

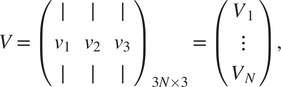

whose 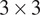 blocks 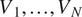 are 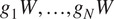 , for some unknown arbitrary 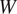 (see^(^^13^ ^)^ for a detailed derivation). In practice, at this point, we replace each 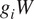 by its closest orthogonal transformation, as described in^(^^14^ ^)^, to improve its accuracy in the presence of noise and discretization errors.

Next, in order to extract an estimate for 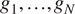 from (14) (i.e., to eliminate 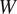 from the estimates in 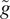 given by (12)), we multiply each 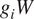 by 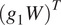 , resulting in
(15)

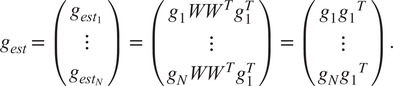

Thus, each 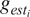 is a rotation, even if 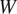 is not. We define 
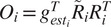
 , and using (9), we get for 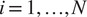



(16)

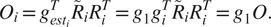

Thus, we have 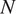 estimates for 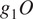 . Equation (4) states that 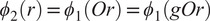 for any symmetry element 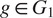 . Therefore, estimating 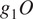 is equivalent to estimating 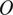 . In order to estimate 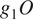 simultaneously from all 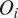 , 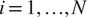 , we search for the rotation 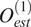 (the superscript will be explained shortly) that satisfies
(17)

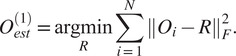

In other words, 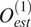 is the “closest” to all the estimated rotations 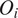 in the least squares sense. To solve (17), let 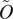 be the 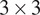 matrix
(18)

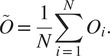

In^(^^15^ ^)^, it is proven that the solution to the optimization problem in (17) is
(19)

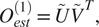

where 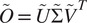 is the singular value decomposition (SVD) of 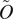 . The algorithm for estimating 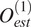 given 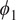 and 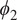 , as described above, is presented in Algorithm 1.

Algorithm 1 Estimating 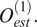

Input: Volumes 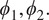

1: Generate random rotations 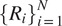

2: Generate from 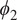 projections 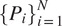 corresponding to the rotations 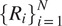 ▷ Eq. (5)

3: Apply Algorithm 2 to each 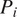 and 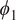 . Denote the resulting rotations by 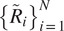

4: **for** 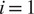 to 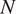 **do**

5: 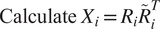 ▷Eq. (10)

6: Construct the matrix 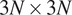

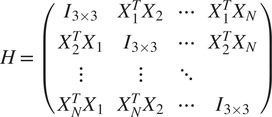

7: Find the three leading eigenvectors 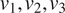 of 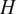

8: Set

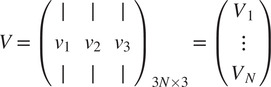

▷ Eq. (14)

9: Compute

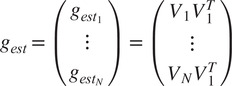

▷ Eq. (15)

10: **for** 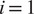 to 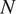 **do**

11: Calculate 
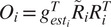
 ▷ Eq. (16)

12: Calculate 
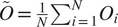
 ▷ Eq. (18)

13: Calculate 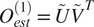 , where 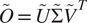 is the SVD of 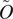 ▷ Eq. (19)

**Output:** 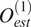 ▷ Estimated rotation

To handle the case where 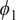 and 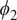 have a different handedness (namely, related by reflection), we can of course apply Algorithm 1 to 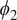 and a reflected copy of 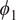 . However, this would roughly double the runtime of the estimation process, as the most time-consuming step in Algorithm 1 is step 3, whose complexity is 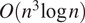 operations for a volume of size 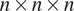 voxels (see Section 5).

Alternatively, it is possible to augment the above algorithm to handle reflections without doubling its runtime. In the case where there is a reflection between 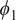 and 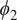 , we need to replace the relation in (2) by the relation
(20)

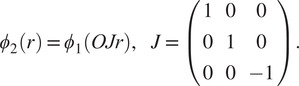

Note that 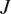 in (20) is a reflection and that 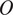 is a rotation. Repeating the above derivation starting from (20) shows that to estimate 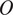 in this case, we can use the same 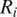 used above and the same estimates 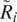 obtained above (steps 1 and 3 of Algorithm 1), but this time we get that 
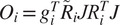
 (compare with (9)). Then, we set 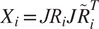 (compare with (10)) and proceed as above, resulting in an estimate 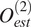 (compare with (17)), which corresponds to the optimal alignment parameters if 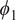 and 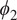 have opposite handedness. Once we have the two estimates 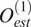 and 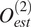 for the alignment parameters between 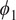 and 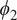 (without and with reflection), we estimate the translation corresponding to each of 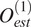 and 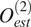 using phase correlation^(^^9^ ^)^ (see Appendix C for details). This results in two sets of alignment parameters (rotation+translation). We then apply both sets of parameters to 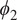 to align it with 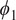 and pick the parameters for which 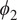 after alignment has higher correlation with 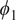 . We denote the estimated parameters by 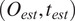 .

## 5. Projection Alignment

It remains to show how to implement step 3 of Algorithm 1, that is, how to find the orientation of a projection 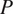 of 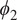 with respect to the coordinate system of 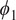 . Mathematically, we would like to solve the equation
(21)

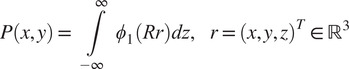

for the unknown rotation 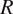 . A brute force approach of testing many candidate rotations in search for the 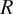 that (best) satisfies (21) is prohibitively expensive, as it requires to compute a projection of 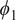 for each candidate rotation (this is essentially projection matching). We therefore take a different approach, whose cost for inspecting each candidate rotation is much lower (in fact requires 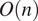 operations to test each candidate rotation for a volume 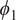 discretized into an array of size 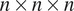 ).

The idea is to generate several projection images from 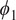 , and then, for each candidate rotation, to check the agreement of the common lines between 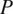 and the projections of 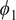 , assuming the orientation of 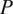 is given by the candidate rotation. We estimate the rotation corresponding to 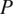 as the candidate rotation that results in the best agreement. We next formalize this method and then analyze its complexity.

We start by considering the case where there is no translation between 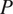 and 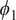 , namely 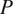 and 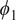 satisfy (21), and our goal is to estimate 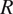 given 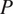 and 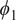 . We generate 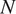 projection images from 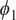 ( 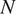 is typically small, see Section 8), denoted 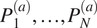 , with rotations 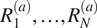 chosen uniformly at random (note that we deliberately reuse the notation 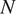 used in Section 4, as explained below). We generate a set of candidate rotations 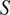 , over which we will search for the solution 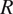 of (21). The set 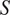 consists of a large number of approximately equally spaced rotations. See Appendix B for a detailed description of the construction of 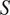 .

We will assume for each candidate rotation 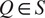 that 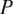 was generated using the rotation 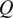 (i.e., we assume that 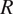 in (21) is equal to 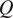 ), compute the mean correlation of the common lines between 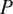 and 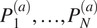 , and choose as an estimate for 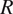 the rotation 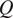 for which the mean correlation is highest. Specifically, for each 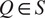 and 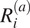 , 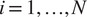 , we compute the direction of the common line between 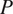 and 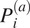 , given by the angles 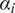 in 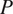 and 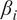 in 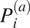 , as explained in Appendix A. The common line property^(^^16^ ^)^ states that if 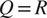 then

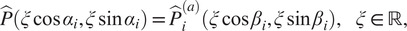

where 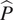 and 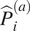 are the Fourier transforms of 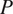 and 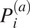 , respectively (see Appendix A for a review of common lines and their properties). We thus define

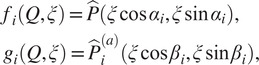

and the cost function
(22)

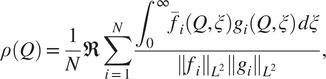

where 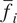 denotes the complex conjugate of 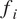 . In other words, 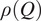 measures how well the common lines induced by 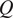 between 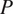 and 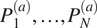 agree. We then set our estimate for 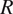 to be

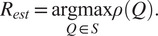

We explore the appropriate value for 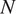 in Section 8.

We now extend the above scheme to the case where 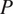 is not centered with respect to 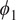 , namely 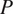 is given by
(23)

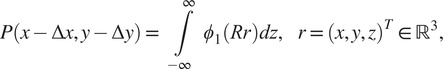

for an unknown rotation 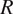 and an unknown translation 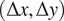 . The idea for estimating 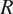 is the same as before, except that the calculation of the common lines should take into account the unknown translation, as we describe next.

We denote the unshifted version of 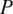 by 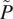 , which is given by
(24)

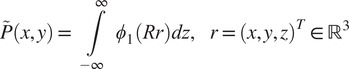

(this is exactly (21), but we repeat it to clearly set up the notation). Then,

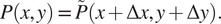

Taking the Fourier transform of both sides of the latter equation, we get that^(^^1^ ^)^
(25)

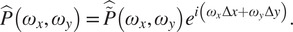

Suppose that the common line between 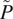 and 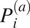 is given by the angles 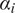 in 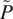 and 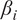 in 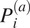 (see Appendix A). By definition of the common line, it holds that

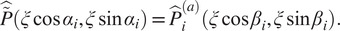

Using (25), we get that

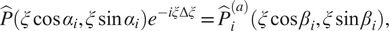

where 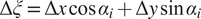 is the one-dimensional shift between the projections along their common line. We assume that this one-dimensional shift is bounded by some number 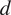 .

Thus, we need to modify our cost function (22) to take into account also the unknown (one-dimensional) phase 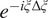 . We therefore define (with a slight abuse of notation in reusing the previous notation for the cost function)

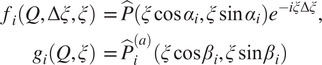

and the cost function
(26)

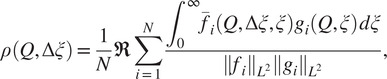

and set our estimate for the solution 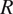 of (23) to be
(27)

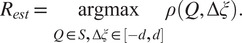

The formula for the angles 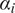 and 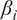 of the common line between 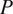 and 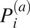 induced by the rotations 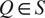 and 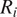 is given in Appendix A. Note that at this point we are only interested in 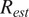 and not in the translation 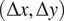 in 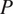 , as the relative translation between 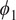 and 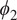 is efficiently determined using phase correlation (see^(^^9^ ^)^ and Appendix C) once we have determined their relative rotation. The algorithm for solving equation (23) is summarized in Algorithm 2.

Algorithm 2 Projection alignment.

**Input:** Projection 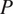 and volume 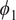 satisfying (23).

1: Generate random rotations 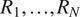

2: Generate from 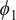 projections 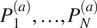 corresponding to the rotations 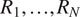

3: Generate candidate rotations 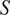 ▷ Appendix B

4: Compute

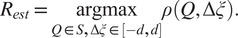

▷ Eqs. 26 and (27)

**Output:** 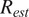 ▷ Estimated rotation

As mentioned above, we use the same 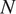 in Sections 4 and 5. While, in principle, the number of projections generated from 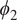 in Section 4 can be different from the number of projections generated from 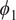 in Section 5, due to the symmetric role of 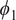 and 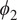 in the alignment problem, there is no reason to consider different values.

## 6. Implementation and Complexity Analysis

Algorithms 1 and 2 are formulated in the continuous domain. Obviously, to implement them, we must explain how to apply them to volumes 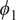 and 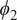 given as three-dimensional arrays of size 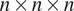 . We now explain how to discretize each of the steps of Algorithms 4 and 5 and analyze their complexity. For simplicity, we use for the discrete quantities the same notation we have used for the continuous ones.

The only step in Algorithm 1 that needs to be discretized is step 2. This step is accurately discretized based on the Fourier projection slice theorem (A.1) using a non-equally spaced FFT^(^^17^ ^,^^18^ ^)^, whose complexity is 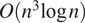 (for a fixed prescribed accuracy). The result of this step is a discrete projection image 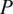 given as a two-dimensional array of size 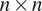 pixels. The remaining steps of Algorithm 4 are already discrete, and since the value of 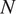 is small compared to 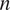 , their complexity is negligible.

We next analyze Algorithm 2. The input to this algorithm is a projection image 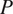 of size 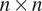 pixels, and a volume 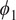 of size 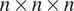 voxels. The algorithm also uses the parameter 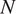 , but since it is a small constant, we ignore it in our complexity analysis. Step 1 of Algorithm 2 requires a constant number of operations. Step 2 is accurately implemented using a non-equally spaced FFT^(^^17^ ^,^^18^ ^)^, whose complexity is 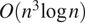 (for a fixed prescribed accuracy). Step 3 is independent of the input volume, and moreover, the set 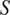 can be precomputed and stored. To implement step 4, we first discretize the interval of one-dimensional shifts 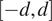 in fixed steps of 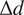 pixels (say, 1 pixel). Specifically, we use the following shift candidates for the optimization in step 4:

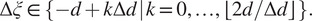

Then, for each 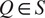 , we compute the angles 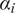 and 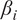 (see Appendix A) and evaluate (26) for the pair 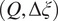 by replacing the integral with a sum. If we store the polar Fourier transforms of all involved projection images 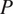 and 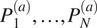 (computed using the non-equally spaced FFT^(^^17^ ^,^^18^ ^)^), each such evaluation amounts to accessing the rays in the polar Fourier transform corresponding to the angles 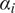 and 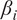 , namely 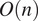 operations. Thus, the total number of operations required to implement step 4 of Algorithm 2 is 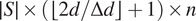 ( 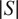 is the number of elements in the set 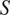 ). Of course, all 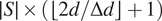 evaluations are independent and can be computed in parallel. Thus, the total complexity of Algorithm 2 is 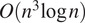 operations for step 2 and 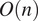 operations for testing each pair 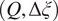 in step 4. Therefore, since the optimization in step 4 is very fast, it is practical to test even a very large set of candidate rotations 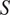 .

Finally, we note that in practice, to further speed up the algorithm, we first downsample the input volumes to size 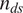 , align the two downsampled volumes, and apply the estimated alignment parameters to the original volumes. We demonstrate in Section 8 that this approach still results in a highly accurate alignment.

To understand the theoretical advantage of the above approach, we compare it to a brute force approach. In the brute force approach, we 1) scan over a large set of rotations and three-dimensional translations, 2) for each pair of a rotation and a translation, we transform one of the volumes according to this pair of parameters, and 3) choose the pair for which the correlation between the volumes after the transformation is maximal. Testing each pair of candidate parameters requires 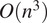 operations (for rotating and translating one of the volumes, and for computing correlation), which amounts to a total of 
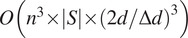
 operations. In other words, testing each candidate rotation and translation is way more expensive than in our proposed method. In our approach, the expensive operation of complexity 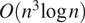 needs to be executed only once per each pair of inputs 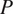 and 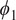 . Moreover, in our approach, the search over shifts is one-dimensional as opposed to the three-dimensional search required in the brute force approach.

## 7. Parameters’ Refinement

In this section, we describe an optional refinement procedure for improving the accuracy of the estimated parameters 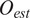 and 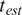 obtained using the algorithm of Section 4.

We define the vector 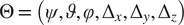 consisting of the six parameters required to describe the transformation between two volumes—three Euler angles ( 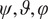 ) describing their relative rotation and three parameters 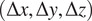 describing their relative translation. We define the operator 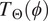 , which applies the transformation parameters 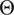 to the volume 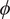 (i.e., 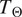 first rotates the volume and then translates it, according to the parameters in 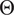 ). Next, for given volumes 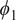 and 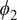 , we denote their correlation by 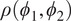 . We are reusing the notation 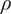 from Section 5, since all occurrences of 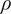 in this paper correspond to a correlation coefficient whose evaluation formula is clear from its arguments. Finally, we define the objective function
(28)

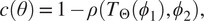

which vanishes for the parameters 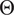 that align 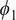 with 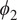 .

To refine 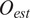 and 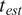 of Section 4, we simply apply the BFGS algorithm^(^^19^ ^)^ to the objective function (28), with an initialization given by 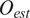 and 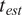 .

## 8. Results

The alignment algorithm (with and without the optional refinement described in Section 7) was implemented in Python and is available online^1^, including the code that generates the figures of this section. A Matlab version of the algorithm is available as part of the ASPIRE software package^(^^20^ ^)^.

As the algorithm uses two parameters—the downsampling 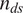 (see Section 6) and the number of reference projections 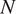 (see Section 4)—we first examine how to appropriately set their values. Then, we examine the advantage of the refinement procedure proposed in Section 7. To show the benefits of our algorithm in practice, we then compare its performance to that of two other alignment algorithms—the alignment algorithm from the EMAN2 software package^(^^10^ ^)^ (implemented in the program e2proc3d) and the FRM algorithm implemented in the Xmipp software package^(^^6^ ^)^. Finally, we examine the performance of the three algorithms using noisy input volumes.

We tested our algorithm on volumes from the electron microscopy data bank (EMDB)^(^^21^ ^)^ with different types of symmetries, whose properties are described in Table 1. All tests were executed on a dual Intel Xeon E5-2683 CPU (32 cores in total), with 768 GB of RAM running Linux. The memory required by the algorithm is of the order of the size of the input volumes. We used 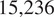 candidate rotations in Algorithm 2 (the size of the set 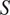 ), generated as described in Appendix B. This set of candidates is roughly equally spaced in the set of rotations 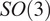 . While it is difficult to characterize the resolution of this set in terms of the resolution of each of the Euler angles, a rough calculation suggests that the resolution in each of the Euler angles is smaller than 5 degrees. We do not use rotations generated by a regular grid of Euler angles, as such a grid is less efficient than our grid, due to the nonuniform rotations generated by a regular grid of Euler angles. For example, discretizing each of the Euler angles to 5 degrees would result in 186,624 rotations, more than an order of magnitude larger than the number of rotations we use.

**Table 1.** Test volumes.

EMDID	Sym	Size ( 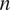 )
2660	C1	360
0667	C2	480
0731	C3	486
0882	C4	160
21376	C5	256
11516	C7	512
21143	C8	256
6458	C11	448
30913	D2	110
20016	D3	384
22462	D4	320
9233	D7	400
21140	D11	324
4179	T	200
24494	I	432

*Note.* Each volume is a three-dimensional array of size 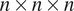 , with 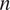 specified on the third column. The symmetry of each volume is given by the second column.

For each test, we generate a pair of volumes 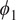 and 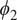 related by a rotation matrix 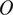 and a translation vector 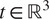 . The translation is chosen at random with magnitude up to 10% of the size of the volume. We denote the alignment parameters estimated by our algorithm by 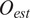 and 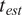 . We evaluate the accuracy of our algorithm by calculating the difference between the rotations 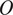 and 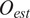 . To that end, we first note that following (4), 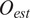 is an estimate of 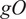 for some arbitrary 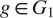 , where 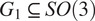 is the symmetry group of 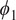 . In order to calculate the difference between 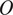 and 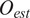 , we have to find the symmetry element 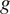 . In our tests, the symmetry group 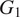 is known (see Table 1), and so we find 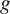 by solving
(29)

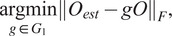

followed by defining 
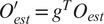
 . Next, the error in the estimated rotation 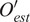 is calculated using the axis-angle representation of rotations as follows. The axis of the rotation 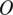 is defined to be the unit vector 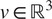 that satisfies 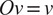 , that is, 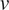 is an eigenvector of 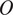 corresponding to eigenvalue 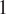 . Similarly, we define the unit vector 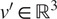 to be the axis of the rotation 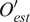 . Then, we calculate the angle between the axes of the rotations as
(30)

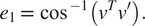

The angle of rotation of the matrix 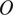 around its axis 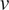 is given by 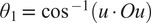 , where 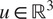 is a unit vector perpendicular to 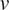 . Similarly, we define 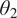 to be the angle of rotation of the matrix 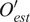 around its axis 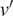 . The error in the rotation angle is then defined as
(31)

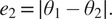

**Figure 2.**
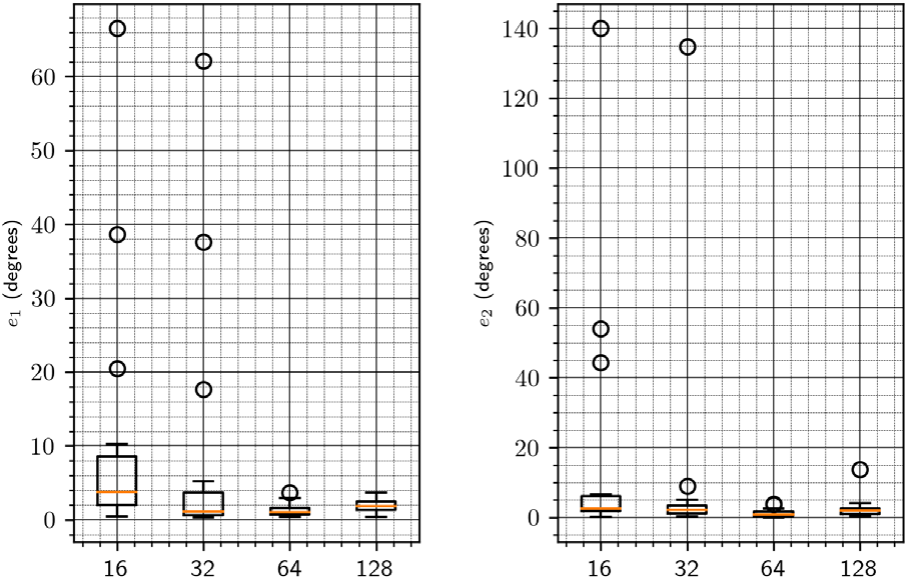
Downsampling parameter versus accuracy of the algorithm. The left figure corresponds to the error 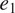 in the rotation axis (see (30)). The right figure corresponds to the error 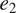 in the rotation angle (see (31)).

**Figure 3.**
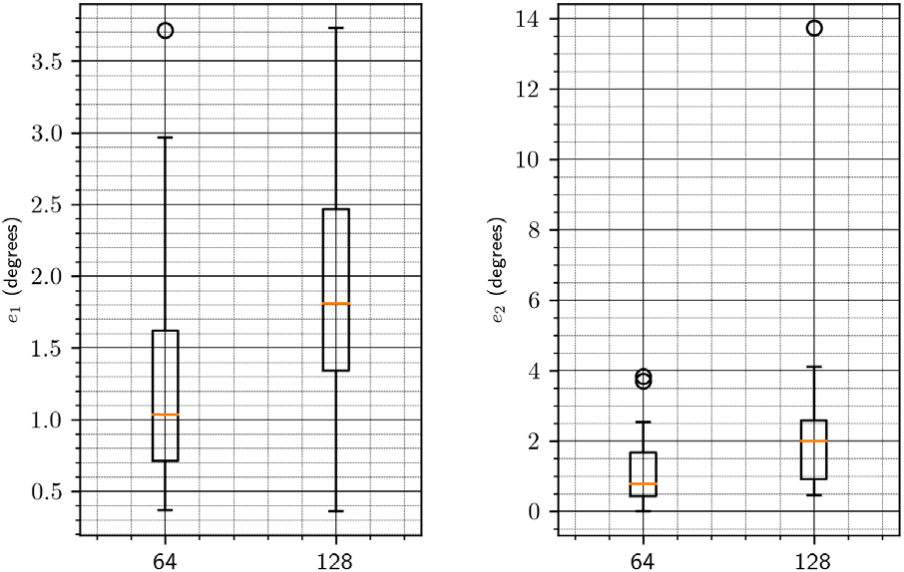
Downsampling parameter versus accuracy of the algorithm, focused on 64 and 128. See Figure 2 for more details.

**Figure 4.**
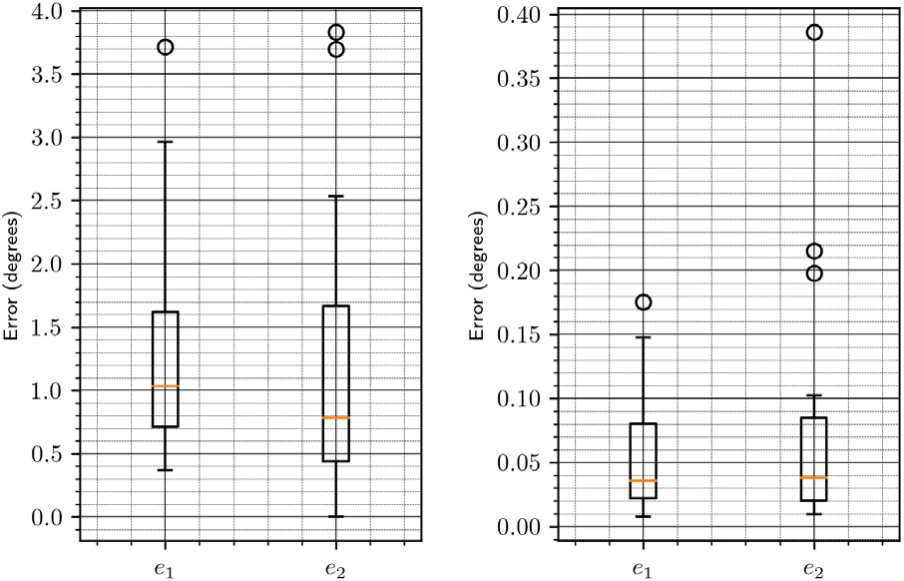
Error without (left figure) and with (right figure) refinement for downsampling to size 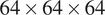 . The error reported in the figure is either 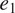 (30) or 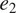 (31), as shown in the 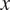 -axis.

**Figure 5.**
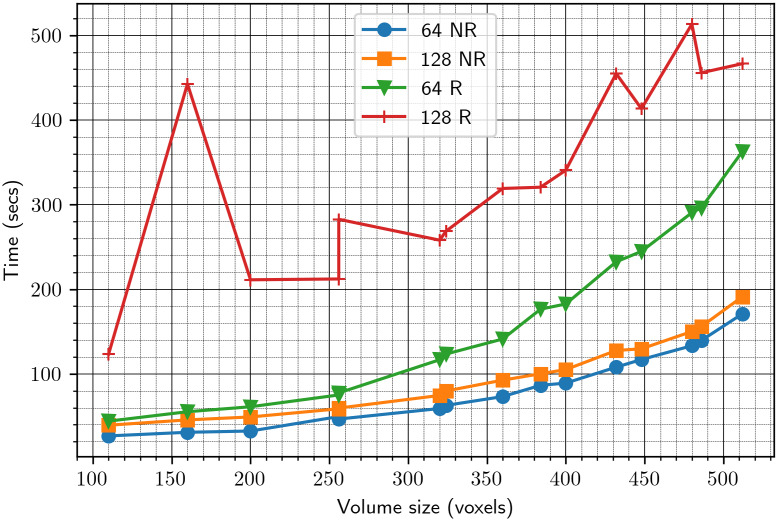
Timing of the alignment algorithm with downsampling to sizes 64 and 128. NR stands for “without refinement”; R stands for “with refinement.”

We start by investigating the appropriate value for the downsampling parameter 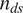 (see Section 6). To that end, for each of the volumes in Table 1, we create its rotated and shifted copy and apply our algorithm with the downsampling parameter equal to 16, 32, 64, and 128 (namely, we actually align downsampled copies of the volumes and then apply the estimated parameters to the original volumes). The results are shown in Figure 2. For each value of downsampling, we show a bar plot that summarizes the results for all test volumes. Note that these results are without the refinement procedure of Section 7. To provide a more detailed information on the chosen downsampling value, we show in Figure 3 only the results for downsampling to sizes 64 and 128. Based on these results, we use a downsampling value of 64 in all subsequent tests. In particular, this value of downsampling results in an accurate initialization of the refinement procedure of Section 7, as shown in Figure 4. As of timing, we show in Figure 5 the timing, without and with refinement, for downsampling to sizes 64 and 128.

**Figure 6.**
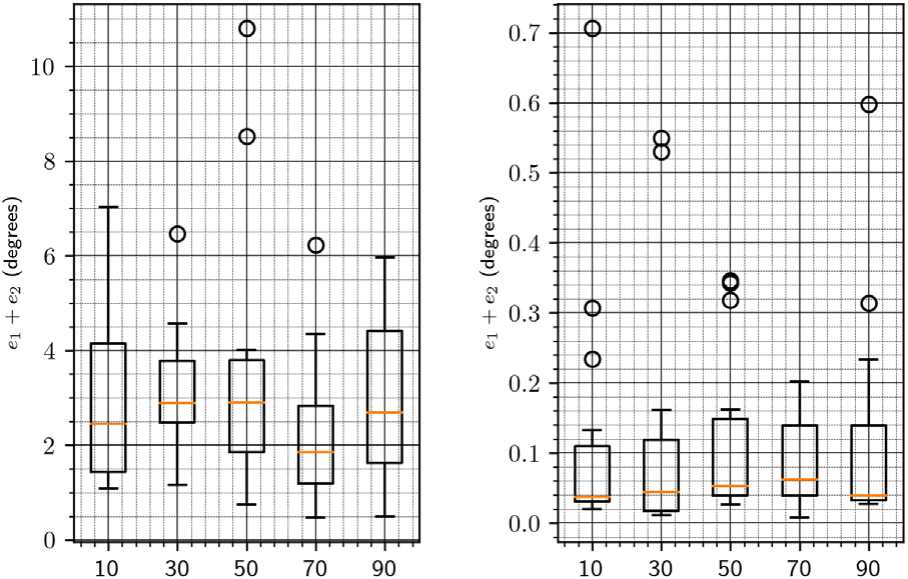
Error versus the number of reference projections 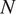 . The left and right figures show the error without and with the refinement procedure of Section 7, respectively. The error reported in this figure is the sum 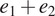 given in (30) and (31).

**Figure 7.**
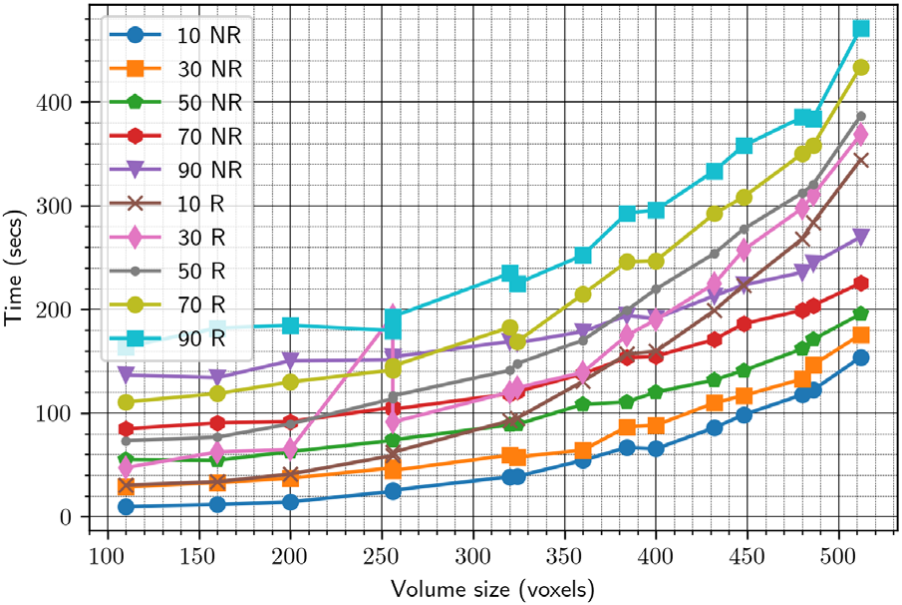
Time versus the number of reference projections.

Next, we wish to determine the number of reference projections 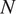 to use in Algorithms 1 and 2. We set the downsampling parameter to 64 and measure the estimation error for different numbers of reference projections. The results are summarized in Figure 6. We also show the timing for different numbers of reference projections, without and with refinement, in Figure 7. Based on these results, we choose the number of reference projections to be 30, as a good compromise between accuracy and speed.

**Figure 8.**
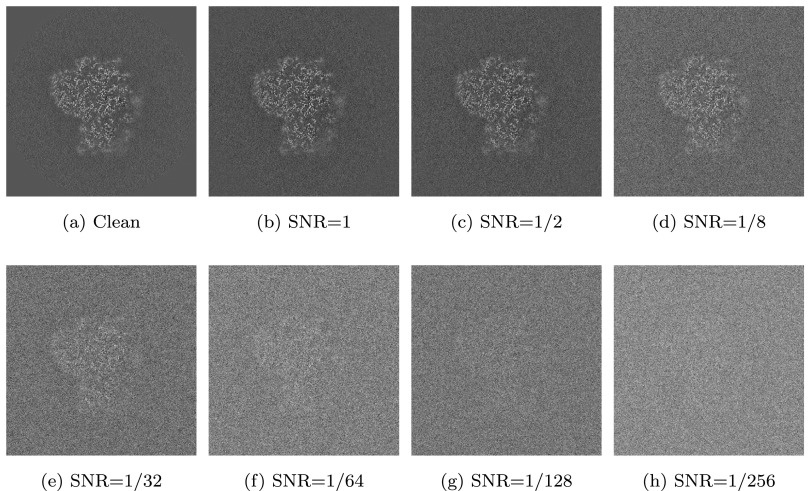
Central slice of the noisy reference volume at different SNRs.

Next, we compare the performance of our algorithm with that of EMAN2’s and Xmipp’s alignment algorithms. The accuracy and timing results are summarized in Tables 2 and 3, respectively. Finally, we demonstrate the performance of the different algorithms for noisy input volumes. To that end, we use as a reference volume EMD 2660^(^^22^ ^)^ from EMDB (of size 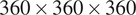 voxels) and create its rotated and translated copy. We add to the reference volume and its rotate/translated copy additive Gaussian noise with SNR (signal-to-noise ratio) ranging from 1 to 1/256. A central slice from the noisy reference volume at different levels of SNR is shown in Figure 8. The accuracy results of all algorithms for the various SNRs are shown in Table 4. The timings of the different algorithms are shown in Table 5.

**Table 2.** Accuracy comparison with EMAN2 and Xmipp.

Sym	EMDID	EMalign(NR)	EMalign(R)	EMAN	Xmipp
C1	2660	2.802	0.094	0.094	5.557
C2	667	3.747	0.223	0.121	7.181
C3	731	8.664	5.131	0.135	48.058
C4	882	1.952	0.041	0.408	0.317
C5	21376	2.949	0.358	0.507	15.445
C7	11516	3.961	0.397	0.153	5.745
C8	21143	1.502	0.536	0.455	2.778
C11	6458	2.825	0.116	0.046	0.314
D2	30913	6.273	0.035	0.425	0.141
D3	20016	3.499	0.075	0.033	1.564
D4	22462	6.016	0.126	0.095	0.251
D7	9233	4.034	0.063	0.029	5.866
D11	21140	3.183	0.042	0.247	0.127
T	4179	1.324	0.556	0.348	6.246
I	24494	3.268	0.030	0.114	0.028
Mean		3.733	0.522	0.214	6.641
Std		1.945	1.288	0.168	12.206

*Note.* The errors reported in this table are the sum 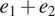 given in (30) and (31). Errors are given in degrees. For EMalign, (NR) corresponds to “without refinement” and (R) to “with refinement.” The two bottom rows show the mean and standard deviation of the error (in degrees) over all experiments.

**Table 3.** Timing comparison with EMAN2 and Xmipp (in seconds).

Sym	EMDID	size	EMalign(NR)	EMalign(R)	EMAN	Xmipp
C1	2660	360	49	130	172	2106
C2	667	480	80	235	354	5812
C3	731	486	85	173	351	5582
C4	882	160	18	55	66	91
C5	21376	256	24	58	155	529
C7	11516	512	78	216	425	6854
C8	21143	256	33	55	120	698
C11	6458	448	54	151	276	3854
D2	30913	110	16	34	55	37
D3	20016	384	41	105	197	2214
D4	22462	320	29	87	201	1095
D7	9233	400	40	124	171	2970
D11	21140	324	35	79	197	1175
T	4179	200	21	59	80	246
I	24494	432	54	158	281	3313

*Note.* For EMalign, (NR) corresponds to “without refinement” and (R) to “with refinement”. The column “size” is the side length of the input volumes.

**Table 4.** Accuracy comparison for noisy input volumes at different SNRs.

SNR	EMalign(NR)	EMalign(R)	EMAN	Xmipp
Clean	4.066	0.143	0.072	0.968
1	3.715	0.145	0.072	0.898
1/2	1.827	0.150	0.072	0.851
1/8	5.733	0.291	0.072	0.728
1/32	5.014	3.318	0.095	0.811
1/64	4.283	0.598	0.105	1.124
1/128	2.727	0.691	0.202	1.177
1/256	4.449	25.089	0.124	1.598
1/512	92.569	97.549	0.288	1.662

*Note.* See Table 2 for more details.

**Table 5.** Timing comparison for noisy input volumes at different SNRs.

SNR	EMalign(NR)	EMalign(R)	EMAN	Xmipp
Clean	33	102	181	2097
1	35	122	176	2091
1/2	39	97	175	2177
1/8	33	105	170	2121
1/32	33	100	155	1991
1/64	38	97	157	2125
1/128	39	113	179	2297
1/256	36	101	163	2092
1/512	39	92	167	2365

*Note.* All timings are given in seconds.

## 9. Discussion and Conclusions

In this paper, we proposed a fully automatic method for aligning three-dimensional volumes with respect to rotation, translation, and reflection. While the parameters of the algorithm can be tuned whenever needed, we showed that the default parameters work very well for a wide range of volumes of various symmetries. We also developed an auxiliary algorithm, which finds the orientation of a volume giving rise to a given projection image (Section 5). This algorithm may serve as a fast and highly accurate substitute to projection matching.

The core difference between our approach and other existing approaches is that our approach is based on common lines between projection images generated from the volumes. The advantage of this approach is that inspecting each candidate rotation is very fast, as it is based on one-dimensional operations on the common lines ( 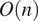 operations for volumes of size 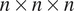 ). We also note that our cost function (26) for identifying the optimal alignment is different than in other algorithms. While the typical cost function used by alignment algorithms is the correlation between the volumes, our cost function is the average correlation of the common lines between projection images of the volumes. These two cost functions are not equivalent, and while in our experiments we have not identified a scenario where one cost function is superior over the other, having tools that are based on different principles may prove beneficial in the future.

From the comparison of our algorithm with the alignment algorithms in EMAN2 and Xmipp, we conclude that our algorithm can be used in one of two modes. If we are interested in fast alignment with good accuracy (average error of 1.9 degrees of the rotation axis, and average error of 1.86 degrees of the in-plane rotation angle, with standard deviations of 1.25 degrees and 1.3 degrees, respectively), we can use our algorithm without the refinement procedure of Section 7. This is appropriate, for example, for visualization, as such an initial alignment is sufficient as an input for high resolution optimization-based alignment algorithms, such as the one in Chimera^(^^3^ ^)^. In such a case, our algorithm is more than 3 times faster than EMAN2’s algorithm (even though our algorithm is implemented entirely in Python), and almost 40 times faster than Xmipp’s algorithm. If we are interested in very low alignment errors, the refinement procedure of Section 7 brings the average errors down to 0.25 degrees for the rotation axis and 0.28 degrees for the in-plane rotation angle (with standard deviations of 0.66 degrees and 0.63 degrees, respectively). In such a case, our algorithm is 80% faster than EMAN2’s and 15 times faster than Xmipp’s.

As of noise robustness, we see from Table 4 that our algorithm performs well down to SNR = 1/128. The algorithms in EMAN2 and Xmipp give very accurate results at even lower SNRs, but at the cost of a much higher running time. As a future research direction, there are several ways to improve the robustness of our algorithm to noise. First, as our algorithm is based on generating projection images of the volumes, it is possible to apply image denoising methods to the projection images. Then, it is possible to incorporate denoising into the common lines matching step (step 4 in Algorithm 2), for example, by denoising the common lines as one-dimensional signals or by incorporating frequency-dependent weights into (26). This is expected to significantly improve the robustness of our algorithm to noise while increasing its running time only slightly.

## Data Availability

Code and documentation of the algorithm are available on https://pypi.org/ and at https://github.com/ShkolniskyLab/emalign.

## Footnotes

1
https://github.com/ShkolniskyLab/emalign

## A. Appendix. Common lines

In this section, we review the Fourier projection slice theorem and its induced common line property. Given a volume  and a rotation matrix  , the projection image of  corresponding to orientation  is given by (5). We identify  (the third column of  ) as the viewing direction of  (see^(^^23^ ^)^). The first two columns  and  of  form an orthonormal basis for a plane in  which is perpendicular to the viewing direction  . Therefore, if  and  are two rotations with the same viewing direction (  ), then the two projection images  and  generated according to (5) look the same up to some in-plane rotation.

The Fourier projection slice theorem relates the two-dimensional Fourier transform of a projection image  to the three-dimensional Fourier transform of  . Let

be the two-dimensional Fourier transform of  , and let

be the three-dimensional Fourier transform of  . The Fourier projection slice theorem^(^^16^ ^)^ states that

(A.1)

where  is defined in (5). (Equation A.1) states that the two-dimensional Fourier transform of each projection image  is equal to a planar slice of the three-dimensional Fourier transform of  . Moreover, it states that this planar slice is the plane  . The Fourier projection slice theorem (A.1) holds, up to discretization errors, also for discrete volumes and their sampled projection images.

From (A.1), we get that any two Fourier transformed projection images  and  with different viewing directions (  are equal to two different planar slices from  . Since there exists a line that is common to both planar slices, the two Fourier transformed images share a common line. We refer to that line as the common line between  and  . We denote the angle that this line makes with the local  -axis of the (Fourier transformed) images  and  by  and  , respectively. Mathematically, the common line property is expressed as^(^^16^ ^)^

implying that the samples of the Fourier transformed images along the common line are equal.

To find an expression for the angles  and  , we consider the unit vector

where  is the cross product between vectors. Define the unit vectors

It can be shown^(^^16^ ^)^ that these vectors satisfy the equation

which implies that  and  can be computed as

from which  and  can be easily extracted.

## B. Appendix. Constructing the set

We generate the set of candidate rotations  by using the Euler angles representation for rotations. Let  be a positive integer, and let  be Euler angles. We construct  by sampling the Euler angles in equally spaced increments as follows. First, we sample  at  points. Then, for each  , we sample  at  points. Finally, for each pair  , we sample  at  points. For each  on this grid, we compute a corresponding rotation matrix  by

where

## C. Appendix. Translation estimation

For completeness, we review the well-known phase correlation procedure for translation estimation^(^^9^ ^)^. Consider two volumes  and  shifted relative to one another, that is,

(C.2)

where  . Our goal is to estimate  .

First, by the Fourier shift property, the Fourier transforms of  and  satisfy

(C.3)

From (C.3), we get^(^^24^ ^)^

(C.4)

since  . Then, since the inverse Fourier transform of a complex exponential is a Dirac delta, we have

(C.5)

Therefore,  is given by

(C.6)

While this appendix is formulated in the continuous domain, the same holds if we replace  and  by their discrete versions sampled on a regular grid and replace the Fourier transform by the discrete Fourier transform.
